# Conservation implications of elucidating the Korean wolf taxonomic ambiguity through whole‐genome sequencing

**DOI:** 10.1002/ece3.10404

**Published:** 2023-08-04

**Authors:** Germán Hernández‐Alonso, Jazmín Ramos‐Madrigal, Xin Sun, Camilla Hjorth Scharff‐Olsen, Mikkel‐Holger S. Sinding, Nuno F. Martins, Marta Maria Ciucani, Sarah S. T. Mak, Liam Thomas Lanigan, Cecilie G. Clausen, Jong Bhak, Sungwon Jeon, Changjae Kim, Kyung Yeon Eo, Seong‐Ho Cho, Bazartseren Boldgiv, Gankhuyag Gantulga, Zunduibaatar Unudbayasgalan, Pavel A. Kosintsev, Hans K. Stenøien, M. Thomas P. Gilbert, Shyam Gopalakrishnan

**Affiliations:** ^1^ Section for Hologenomics, The Globe Institute University of Copenhagen Copenhagen Denmark; ^2^ Center for Evolutionary Hologenomics, The Globe Institute University of Copenhagen Copenhagen Denmark; ^3^ Department of Biology University of Copenhagen Copenhagen Denmark; ^4^ Clinomics Inc. Ulsan Korea; ^5^ Korean Genomics Center Ulsan National Institute of Science and Technology Ulsan Korea; ^6^ Department of Biomedical Engineering, College of Information‐Bio Convergence Engineering Ulsan National Institute of Science and Technology Ulsan Korea; ^7^ Personal Genomics Institute Genome Research Foundation Osong Korea; ^8^ Department of Animal Health & Welfare Semyung University Jecheon Korea; ^9^ Natural History Museum Kyungpook National University Gunwi Korea; ^10^ Laboratory of Ecological and Evolutionary Synthesis National University of Mongolia Ulaanbaatar Mongolia; ^11^ Institute of Biology Mongolian Academy of Sciences Ulaanbaatar Mongolia; ^12^ Institute of Plant and Animal Ecology, Urals Branch of the Russian Academy of Sciences Yekaterinburg Russia; ^13^ Ural Federal University Ekaterinburg Russia; ^14^ NTNU University Museum Norwegian University of Science and Technology Trondheim Norway; ^15^ University Museum Norwegian University of Science and Technology Trondheim Norway; ^16^ Bioinformatics, Department of Health Technology Technical University of Denmark Lyngby Denmark

**Keywords:** *Canis lupus*, population genomics, taxonomic ambiguity, wolf conservation genetics, wolf population structure

## Abstract

The taxonomic status of the now likely extirpated Korean Peninsula wolf has been extensively debated, with some arguing it represents an independent wolf lineage, *Canis coreanus*. To investigate the Korean wolf's genetic affiliations and taxonomic status, we sequenced and analysed the genomes of a Korean wolf dated to the beginning of the 20th century, and a captive wolf originally from the Pyongyang Central Zoo. Our results indicated that the Korean wolf bears similar genetic ancestry to other regional East Asian populations, therefore suggesting it is not a distinct taxonomic lineage. We identified regional patterns of wolf population structure and admixture in East Asia with potential conservation consequences in the Korean Peninsula and on a regional scale. We find that the Korean wolf has similar genomic diversity and inbreeding to other East Asian wolves. Finally, we show that, in contrast to the historical sample, the captive wolf is genetically more similar to wolves from the Tibetan Plateau; hence, Korean wolf conservation programmes might not benefit from the inclusion of this specimen.

## Graphical abstract

To investigate the likely extirpated Korean wolf genetic affiliations, we sequenced and analysed the genomes of a historical Korean wolf, and a captive wolf. Our results indicated that the Korean wolf bears similar genetic ancestry to other regional East Asian populations, and identified regional patterns of wolf population structure and admixture in East Asia with potential conservation consequences on a regional scale.

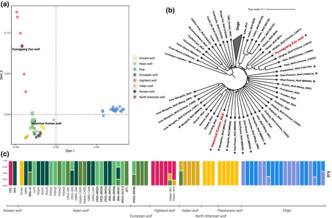

## 1 INTRODUCTION

Species and subspecies ambiguity is a common problem in canid taxonomy due to their wide and continuous distribution, their long‐distance mobility across topographic barriers, the extended gene flow among populations, and even, hybridisation among closely related species (Gopalakrishnan et al., 2018; Nowak, 1995, 2010; Pilot et al., 2006; Shrotriya et al., 2012; Wayne & Vilà, 2003). A specific case of taxonomic ambiguity that has not been properly resolved is represented by the likely extirpated wolf populations from the Korean Peninsula.

The Korean wolf's geographical distribution spanned the entire Korean Peninsula until the middle of the last century. However, its population was severely impacted by the Japanese incursion into Korea (1910–1945) and the subsequent industrialisation programme. The remaining populations were finally extirpated from South Korea during the late 20th century. The last individuals were recorded during the 1990s around Paektu Mountain, located at the border between North Korea and China (Jo et al., 2018). At present, wolves are considered extirpated from the entire Peninsula according to the Red Data Book of South Korea (National Institute of Biological Resources, 2012). However, there is very limited information about the conservation status of wolf populations in North Korea.

Korean wolf populations have been subject to several taxonomic changes since the late 19th century. At first, Korean wolves were included in the original definition of *Canis chanco* proposed by Gray (1863) together with populations from the Chinese Tartary (Central China, Northern China, and Mongolia). Later, Mivart (1890) suggested that *C. chanco* and the wolves from Tibet, *Canis laniger* Hogdson, 1847, were probably the same taxonomic group, but also, that both forms were varieties of the common grey wolf, *Canis lupus* Linnaeus, 1758, changing them to a subspecies category. The status of Korean wolf populations was not revised until 1923, when the Japanese zoologist Yoshio Abe suggested that the Korean wolves were morphologically different enough from continental populations to be considered a different species: *Canis coreanus* (Abe, 1923). The British zoologist Reginald Pocock rejected such a proposal, including the Korean wolves in the form *Canis lupus laniger* (synonym of *Canis lupus chanco*) together with populations from China, Kashmir, and Tibet (Pocock, 1935).

In 2005, Wozencraft reasserted the subspecies *C. l. chanco*, including *C. coreanus* Abe, 1923 as one of its synonyms. Importantly, the *C. l. chanco* subspecies by itself is a well‐known case of taxonomic ambiguity that has been frequently discussed (cf. Aggarwal et al., 2003, 2007; Joshi et al., 2020; Shrotriya et al., 2012). At that time, the *C. l. chanco* subspecies distribution was considered to cover a large area of central and east Asia, as suggested by Sokolov and Rossolimo (1985). Later, genomic data confirmed a high degree of genetic divergence between Himalayan and Tibetan Plateau wolf populations (Aggarwal et al., 2003), as well as their unique adaptations to high altitude environments (Werhahn et al., 2018). Then, in 2019, the IUCN/SSC Canid Specialist Group recommended using the name *C. l. chanco* for those populations restricted to the Himalayan range and the Tibetan Plateau (Alvares et al., 2019). Consequently, the remaining Asian wolf populations included before in *C. l. chanco* were implied to belong to *C. l. lupus*. Although these taxonomic changes included the Korean wolf population, its validity has yet to be tested using genomic data.

The first time that a Korean wolf was included in a molecular analysis was by Ishiguro et al. (2009, 2010), where the mitochondrial control region (751 bp) was used to study the extinct Japanese wolf (*C. l. hodophilax*) and Ezo wolf from Hokkaido (*C. l. hattai*). The Korean wolf sample used in that study is described as a *C. l. chanco* and it was collected from the Hasebe Collection at the University of Tokyo Museum. In the results presented by Ishiguro et al. (2009, 2010) the Korean wolf seems to be closely related to Chinese and Mongolian wolves, as would be expected based on geography. However the variation in the mitochondrial control region has limited resolution, hence additional work including autosomal variation is needed to truly evaluate the affiliation of original Korean wolves.

Here, we generated whole‐genome sequencing data from a captive wolf, putatively North Korean, as well as a historical wolf from South Korea, to an average depth coverage of 25.29× and 7.25× respectively (Table S1). Additionally, we resequenced 10 wolves from poorly characterised populations in Russia, Mongolia and Kazakhstan, and a dead Mongolian captive wolf from Zoo Zürich to an average depth coverage of 5–7×, to obtain a better representation of Asian wolf populations (Table S1). The historical Korean wolf (HKW) genome was obtained from a female specimen housed at the Kyungpook National University Natural History Museum, Gunwi‐gun, South Korea (Figure S9). This individual dates to the beginning of the 20th century (before 1945), and given its provenance, it likely represents one of the last wolf populations in South Korea. The modern genome was obtained from a female captive wolf, originally located in the Pyongyang Central Zoo, North Korea, and subsequently transferred to Seoul Grand Park in 2005. Her wild origin is ultimately uncertain. Using these samples, we aimed to resolve the taxonomic ambiguity of the Korean wolf, searching for the most closely related continental wolf population and analysing its level of genetic differentiation. Moreover, we endeavour to track the potential origin of the captive wolf to confirm if its lineage came from inside or outside of the Korean Peninsula.

## 2 MATERIALS AND METHODS

### 2.1 Description of wolf samples

Three subsamples of the HKW were collected from the skin of a female mounted specimen housed at the Kyungpook National University Natural History Museum, Gunwi‐gun, South Korea (Figure S9). Blood samples were taken from a female captive wolf (PZW) from Seoul Grand Park that was transferred from the Pyongyang Central Zoo, North Korea on April 14, 2005, through animal exchange between South and North Korea. Finally, a tissue sample was obtained from a dead captive Mongolian wolf (A2) (putatively *C. l. chanco*, Local ID: ZURICH/A70094) from Zoo Zürich.

Muscle samples from modern specimens 750021A and 750115A were provided by the Yekaterinburg Museum, Russia. A further skin sample (MW486) and extracts (MW524, MW536, and MW538) were also collected from Russian wolf populations and provided by collaborators. Samples from muscle, cheek tissue, and skin corresponding to MW561, MW574, and MW588 were obtained from contemporary Mongolian wolves.

These data were generated as part of a broader study funded by the Norwegian Environment Agency that aims at looking at the relationship of modern and historic Norwegian wolves to other Eurasian wolves (Stenøien et al., 2021).

### 2.2 Laboratory processing of modern samples

DNA from modern wolf PZW (SGP‐824) was extracted using the DNeasy Blood & Tissue kit (Qiagen), according to the manufacturer's recommendations. DNA quality was assessed by running 1 μL on the Bioanalyzer system (Agilent) to ensure size and analysis of DNA fragments. The concentration of DNA was assessed using the dsDNA BR assay on a Qubit fluorometer (Thermo Fisher Scientific). DNA was converted into double stranded blunt‐end libraries with BGI‐specific adapters (Mak et al., 2017) using the BEST protocol (Carøe et al., 2018). Libraries were sequenced on a BGISeq 500 plataform using 100 base pair paired‐end read chemistry.

DNA from a modern Mongolian wolf (A2) was extracted using a DNeasy Blood & Tissue Kit (Qiagen), following the manufacturer's protocol. DNA was converted into double‐stranded blunt‐end libraries with Illumina‐specific adapters REF using the NEBNext DNA Sample Prep Master Mix Set 2 (E6070S; New England Biolabs Inc.), following the manufacturer's protocol. Libraries were sequenced on a Illumina HiSeq 2500 platform using 100 base pair paired‐end read chemistry.

DNA from modern wolves samples (MW486, MW524, MW536, MW538, MW561, MW574, MW588, 750021A, 750115A) was extracted and prepared for sequencing in the DNA laboratories at the Globe Institute, University of Copenhagen, using a Kingfisher duo prime extraction robot. DNA extracts were sent to BGI Denmark for library building and sequencing. The samples were sequenced in a DNBseq platform in PE150 mode.

### 2.3 Laboratory processing of historical samples

The HKW sample was processed under strict clean laboratory conditions at the Globe Institute, University of Copenhagen. Three tissue samples were placed into three Eppendorf tubes—1, 2, and 3—and washed with diluted bleach, ethanol and ddH_2_O, following Boessenkool et al. (2017). The material was processed following Gilbert et al. (2007) DNA extraction protocol. Additional treatment with phenol chloroform was performed following Carøe et al. (2018). The supernatant was then purified using a modified PB buffer and eluted using two washes in 18 μL buffer EB (Qiagen)—with 3 min of incubation time at 37°C (Dabney et al., 2013). The concentration of each extract was checked on a Qubit (ng/μL). BGI libraries were built using 10–20 μL of extract in a final reaction volume of 50 μL following the Santa Cruz Single Stranded protocol (Kapp et al., 2021), and using the “single‐tube” library building protocol BEST (Carøe et al., 2018). Library index amplifications were performed using PfuTurbo Cx HotStart DNA Polymerase (Agilent Technologies) in 50 μL PCR reactions that contained 5 μL of purified library, 0.1 μM of each forward (BGI 2.0) and custom made reverse primers (Mak et al., 2017). The PCR cycling conditions were: initial denaturation at 95°C for 2 min followed by 20 cycles of 95°C for 30 s, 60°C for 30 s, and 72°C for 2 min, and a final elongation step at 72°C for 10 min. Amplified libraries were then purified using 1.8× ratio of MagBio beads to remove adaptor dimers and eluted in 30 μL of EBT (Qiagen) after an incubation for 5 min at 37°C. Amplified libraries were sequenced at Clinomics Inc. with a SE100 mode, and at BGI China with a SR100 mode.

### 2.4 Dataset

The dataset used for this study includes 74 canid whole‐genomes: One Andean fox (*Lycalopex culpaeus*) (Auton et al., 2013), two coyotes (*Canis latrans*) (Gopalakrishnan et al., 2018; vonHoldt et al., 2022), 51 wolves (*C. lupus*) (Fan et al., 2016; Freedman et al., 2014; Hennelly et al., 2021; Niemann et al., 2021; Ramos‐Madrigal et al., 2021; Sinding et al., 2018, 2020; vonHoldt et al., 2016; Wang et al., 2013, 2016; Zhang et al., 2014), and 20 dogs (Auton et al., 2013; Decker et al., 2015; Freedman et al., 2014; Kim et al., 2012; Kolicheski et al., 2017; Lindblad‐Toh et al., 2005; Marchant et al., 2017; Marsden et al., 2016; Metzger et al., 2017; Sinding et al., 2020; Wang et al., 2016) Among the wolf samples, 40 are reference genomes mainly representing Asian populations; five correspond to Pleistocene wolves from Siberia, 10 correspond to wolves resequenced for this study representing wolf populations in Asia, and two are the Korean wolves (a modern captive wolf [PZW] from the Seoul Grand Park in South Korea, and a 20th century mounted Korean wolf [HKW] from the Kyungpook University Museum; Figure S9). Finally, all dog genomes included here have been previously published and they were selected to mainly represent Asian breeds (Table S2).

### 2.5 Data processing

Sequence reads obtained from BGI were mapped to the wolf reference genome (Gopalakrishnan et al., 2017) using PALEOMIX v.1.2.13.3 BAM pipeline (Schubert et al., 2014). The wolf reference genome was generated from a highly inbred Swedish wolf, which mitigates issues with reference mapping bias. In brief, adapter trimming was performed using AdapterRemoval v.2.2.0 (Schubert et al., 2016) and only reads with a minimum length of 25 bp were kept. Trimmed reads were mapped to the reference genome using BWA v.0.7.16a backtrack algorithm (Li & Durbin, 2009), disabling the use of a seed parameter. PCR duplicates were identified and removed using Picard MarkDuplicates (Broad Institute, 2023) and, finally, local realignment around indels was performed using GATK v.3.8 3 IndelRealigner module (McKenna et al., 2010).

To evaluate the substitution patterns in the sequences of the HKW DNA, we used mapDamage v.2.0.9 (Jónsson et al., 2013). The aligned sequences resulting from the mapping process were used as input with the default parameters. This step allowed us to authenticate the historical condition of the analysed sample. The HKW DNA sequences showed relatively low levels of fragmentation and deamination patterns typical of ancient DNA damage (Figure S1).

### 2.6 Multidimensional scaling plot

We performed single nucleotide polymorphism (SNP) calling by randomly sampling a read for every site for all the samples in our dataset using the option ‐doHaploCall from ANGSD v.0.931 (Korneliussen et al., 2014). This option allows sampling a random read from each site and each sample instead of performing genotype calling, which allows the incorporation of samples sequenced to low‐ to medium‐depth of coverage. We used the following parameters: doCounts 1 ‐minMinor 2 ‐maxMis 7 ‐C 50 ‐baq 1 ‐uniqueOnly 1 ‐remove_bads 1 ‐only_proper_pairs 1 ‐skipTriallelic 1 ‐doMajorMinor 1. Additionally, bases with quality lower than 20 and mapping quality lower than 30 were discarded. Transitions were removed to avoid aDNA damage that could be found in historical samples. A MAF filter of 0.01 was applied given a final SNPs dataset of 3,284,758 transversion sites. Finally, we restricted the analysis to the scaffolds with at least 1 Mb in size.

Using the previously mentioned SNPs dataset, we estimated pairwise identity‐by‐state (IBS) genetic distances between the samples, and generated a multidimensional scaling (MDS) analysis using Plink v.1.90 (Chang et al., 2015). A first MDS was estimated including all samples except for the outgroups, and a second MDS was estimated restricting to wolves.

### 2.7 Admixture analysis

In order to estimate the ancestry components in the HKW and the wolf (PZW) from South Korean Zoo included in our dataset, we used the previously described SNPs panel and ADMIXTURE v.1.3.0 (Alexander et al., 2009). Outgroups were excluded from this analysis. We ran ADMIXTURE assuming 2–10 (*K*2–*K*10) ancestry components. Ten replicates for each *K* value were performed, and the replicate with the best likelihood value was selected. R library Pophelper v.2.3.1 (Francis, 2017) was used to visualise the admixture plots.

### 2.8 Neighbour‐joining tree

A Neighbour‐joining tree (NJ) was estimated based on an IBS distance matrix constructed in Plink v.1.90, using the function ‐‐distance‐matrix. The same SNPs panel described above was used as input to construct the distance matrix. The tree was estimated using R library *ape* (Paradis & Schliep, 2019) and Interactive Tree Of Life (iTOL) v.4 online tool (Letunic & Bork, 2019) was used to visualise it.

### 2.9 Maximum likelihood nuclear genome phylogeny

A maximum‐likelihood (ML) nuclear genome phylogeny was inferred to explore the evolutionary relationships among the samples from our dataset. The Andean fox and two coyotes were included as outgroups. For each genome, ANGSD v.0.931 was used to generate genomic consensus sequences using the wolf reference genome (“‐dofasta2” option). Then, 1000 independent phylogenetic trees were estimated with RAxML‐ng v.0.9.0 (Kozlov et al., 2019) under the GTR + G evolutionary model, using 1000 random regions of 5000 bp. All gene trees were concatenated to generate a species tree using ASTRAL‐III (Zhang et al., 2018), which was visualised using the Interactive Tree Of Life (iTOL) v.4 online tool (Letunic & Bork, 2019).

### 2.10 TreeMix

To explore admixture patterns in our HKW, the software TreeMix v.1.13 (Pickrell & Pritchard, 2012) was used to estimate an admixture graph. We included a subset of samples comprising the HKW, the Pyongyang Zoo wolf (PZW), Asian wolves that appeared close to the HKW based on the *f*_3_‐statistics and the estimated NJ‐tree, as well as wolves representing different clades on the tree (Portuguese, Xinjiang‐CAN30, Indian‐BH6 and Tibet‐CAN9A), and Asian dog breeds, excluding other dog breeds to avoid the estimation of migration events among the different dog lineages. The final subset consisted of 16 wolves, 12 dogs and 2 coyotes used as outgroups. TreeMix was run using the previously created SNPs panel, restricting the analysis to sites without missing data (1,904,538 sites), estimating 0–5 migration events (‐*m*) and grouping SNPs in windows of 500 (‐*K*). Ten replicates were performed for each migration event and the one with the best likelihood was chosen. The final results were plotted on R using TreeMix script plotting_funcs.R.

### 2.11 Outgroup *f*_3_‐statistics

We calculated outgroup *f*_*3*_‐statistics using the qp3pop tool from ADMIXTOOLS v.5.1 package (Patterson et al., 2012) to measure the amount of share drift between two populations since their common ancestor. For this analysis we used the coyotes as the outgroup. We estimated the shared drift between all our samples and the two Korean wolves to find their most closely related population. Higher values indicate a closer relationship due to shared drift between the two populations in the test.

### 2.12 *D*‐statistics

We used our SNPs panel to run *D*‐statistics, as implemented in qpDstat from ADMIXTOOLS v.5.1 (Patterson et al., 2012) to evaluate the possibility of admixture between the historical Korean wolf and dog breeds, or the captive wolf from the Seoul Grand Park (Pyongyang Zoo wolf) and dog breeds. For a given test in the form *D* (Outgroup, A; B, C), if the obtained *D*‐statistic significantly deviates from 0, it suggests possible gene flow between A and B (*D* < 0) or A and C (*D* > 0). In all the analyses, the Andean fox was used as outgroup. Deviation from 0 was considered statistically significant when the *Z*‐score was below −3 or above 3. The significance of the test was assessed using a weighted block jackknife procedure over 1 Mb blocks.

We evaluate the following tests for both the HKW and the captive PZW:

- *D* (Outgroup, Korean wolf; Tibetan Mastiff, Dogs);
- *D* (Outgroup, Pyongyang Zoo wolf; Tibetan Mastiff, Dogs);
- *D* (Outgroup, Eurasian wolves; Tibetan Mastiff, Shar Pei);
- *D* (Outgroup, Dogs; Portuguese wolf, Korean wolf);
- *D* (Outgroup, Dogs; Portuguese wolf, Pyongyang Zoo wolf).

### 2.13 Genotype imputation

In order to have a more robust dataset for analyses affected by missingness, we imputed the genotypes with Beagle v.5.4 (Browning et al., 2018). First, we generated a SNPs panel using 28 high coverage samples (>7×) representing most of the wolf genomic diversity around the world (PZW, HKW, Yellowstn, Alaska1, Alaska2, Portugal, MNG‐A2, CRUS‐GW1, ERUS‐GW2, WRUS‐GW3, InMNG‐GW4, LionN, Qinghai, Shanxi1, Xinjiang1, Xinjiang2, Xinjiang3, Xinjiang4, Qinghai‐CAN11, Qinghai‐CAN16, Xinjiang‐CAN24, Xinjiang‐CAN30, Tibet‐CAN32, InMNG‐CAN6, InMNG‐CAN7, Tibet‐CAN9A, IndianW‐KZ, India‐BH126) (Table S2). SNP and indel variants per sample were detected using the function HaplotypeCaller in GVCF mode as implemented in GATK v.4.2.0.0 (McKenna et al., 2010). Individual gVCF files were merged using GATK's GenomicsDBImport function, restricting the process to the scaffolds with at least 1 Mb in size. Subsequently, the GATK function GenotypeGVCFs was used to preform joint genotyping, followed by the SelectVariants function to select only SNP variants. Lastly, the final VCF was filtered with VCFtools v.0.1.14 (Danecek et al., 2011) using the parameters ‐‐remove‐indels ‐‐max‐missing 0.9 ‐‐minQ 30 ‐‐minDP 5 ‐‐maxDP 100. Percentages of missingness were estimated with VCFtools v.0.1.14, showing 50% of missingness in the HKW genome.

We then performed genotype calling at the sites of the SNP panel for all samples (*N* = 71) using BCFtools v.1.12 (Danecek & McCarthy, 2017). Beagle v.5.4 was then used for imputation with default parameters using a sliding window size of 40.0 cM and an overlap of 2 cM between adjacent windows. After applying a MAF filter of 0.05, we obtained a final dataset of 4,036,734 transversion sites. In order to evaluate the accuracy of the estimated imputation, we conducted an MDS analysis, as explained in the MDS plot section, utilising the imputed genotypes. The MDS plot generated from this analysis exhibited the same structure as the one obtained from the SNP dataset prior to the imputation process (Figure S8A).

### 2.14 Heterozygosity based on genotype‐likelihoods

We used picard‐tools to downsample 32 wolf genomes to the same number of mapped reads (~58 million mapped, available for the lowest coverage sample). We estimated genotype likelihoods for each of the samples using ANGSD at the 4,036,734 SNP sites used for estimating heterozygosity with the imputed dataset. Reads with mapping quality below 30, bases with quality below 20 and sites with depth of coverage lower than 3 were not considered. Genotype‐likelihoods were used to estimate the site frequency spectrum (SFS) for each sample independently using ANGSD realSFS (Korneliussen et al., 2014). The per‐sample SFS was used as a relative heterozygosity estimate for each sample. We observe a similar pattern to that obtained from the heterozygosity estimates using the imputed dataset (Figure S8B).

### 2.15 Heterozygosity per windows and inbreeding coefficient

Imputed genotypes were used to calculate heterozygosity per window using Plink v.2.3.8. Window sizes of 1 Mb were defined on the first 704 scaffolds longer than 1 Mb. The Plink function *‐‐sample‐counts* was implemented to estimate the number of heterozygous sites for each window, and finally, heterozygosity per window ratio was calculated (heterozygous sites/total sites) and the results were visualised using R. To estimate the inbreeding coefficient, we implemented the Plink function *‐‐het* on the same imputed genotype dataset.

## 3 RESULTS

### 3.1 Korean wolves' population affinities

**FIGURE 1:**
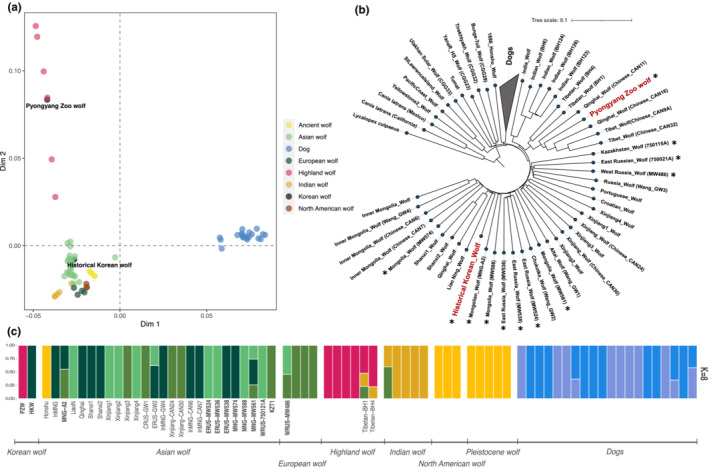
Korean wolf genomic affinities and population structure. (a) Multidimensional scaling plot using genome‐wide data and including all dogs and wolves in the dataset. (b) Distance‐based Neighbour‐joining (NJ) tree showing the placement of the historical Korean wolf (HKW) and the captive Pyongyang Zoo wolf (PZW). (c) Admixture plot assuming 8 ancestry components (*K*), showing that the HKW and the captive PZW cluster with different groups. Each bar corresponds to a different genome. The colours indicate the inferred ancestry components and their proportions. Asterisks next to sample names in the NJ tree as well as bold names in the admixture plot indicate the de novo resequenced wolf genomes.

To explore the population structure in our dataset and assess how the PZW and the HKW relate to wolf and dog populations worldwide, we used a MDS analysis. The results demonstrate that the samples cluster in three main groups: Dogs, highland wolves (represented by the wolves from the Tibetan Plateau), and the remaining wolves in our dataset (Figure 1a). Dimension 1 separates dogs and wolves, while dimension 2 separates the highland wolves from the rest of the wolf populations. Notably, the two Korean samples are not placed together, but rather the HKW clusters in the main wolf cluster near to the East Asian wolves, while the PZW clusters together with highland wolves from the Tibetan Plateau.

To elucidate in more detail the internal population structure among the wolves, we performed a second MDS analysis excluding all dog samples. In this case, dimension 1 separates highland wolves from the rest of the wolf samples and dimension 2 separates Indian wolves (Figure S2). The third group of wolves observed in the upper right corner of the plot includes the North American, ancient Siberian, European and Asian wolves. The internal structure of this last‐mentioned group of wolves resembles the geographical distribution of the Eurasian samples from east to west separated along dimension 2. Consistent with our previous observation, we observe that the PZW and the HKW samples show affinities to different populations. Specifically, the PZW is placed close to the Qinghai‐CAN11 wolf from central China, and the HKW close to Mongolian and north Chinese wolves. In contrast, the other 10 resequenced wolf genomes are placed according to their geographical origin. East Russian wolves (MW536, MW538, and MW524) cluster together with other wolves from the same region, and the Mongolian wolves (MW561, MW588, MW574, and MGN‐A2) cluster with other Mongolian or Inner Mongolian wolves. Wolves from west Russia (MW486) and west Kazakhstan (KZT1) cluster near European wolves, except for the west Russian wolf 750021A, which appeared between the European wolf cluster and west Chinese, central Russian and some ancient Siberian wolves.

We then performed a clustering analysis using ADMIXTURE (Alexander et al., 2009), to estimate individual ancestries assuming 2–10 ancestry components (*K*). When eight ancestry components were estimated, two different dog clusters were recovered. The remaining clusters include (1) ancient wolves and North American wolves together, (2) Indian wolves, (3) highland wolves, and finally, (4) three Eurasian wolf clusters. The results again reveal that the two Korean samples derive from clearly distinct sources, with the HKW clustering principally with wolves from China, while the PZW clusters with the highland wolves (Figure 1c). Across the different estimated ancestry components the same pattern can be observed, where the PZW clusters with wolves from Tibet and Qinghai, and the HKW clusters with East Asian wolves, mainly from Mongolia and northern China. Similar to the MDS results, the rest of the wolves show a clustering pattern that resembles their geographical distribution, including the resequenced Asian wolf genomes (Figure S3).

To investigate the phylogenetic relationships of the PZW and HKW, we performed a distance‐based NJ tree and the ML phylogeny based on 1000 concatenated trees (Figure 1b; Figure S4). In both approaches, we recover the same clades, although the relationships of the basal branches differ. In the NJ tree, the main clades correspond to North American wolves, Pleistocene wolves, Eurasian wolves, and dogs (Figure 1b). Wolves are further subdivided into Indian wolves, highland wolves (including PZW), and European and Asian wolves, with HKW placed at the base of the clade with the Liao Ning, Qinghai, and Shanxi wolves (Figure 1b). Overall, the ML phylogeny recovered similar groupings. However, while the North American wolves represent the most basal clade in the NJ tree, the Indian wolves are placed as the most basal clade in the ML phylogeny. These results are consistent with previous studies showing that reconstructing the basal relationships between major wolf clades is challenging due to gene flow between lineages (e.g., Hennelly et al., 2021). In both phylogenetic reconstructions, the PZW is placed close to highland wolves from Qinghai with a high bootstrap support. In contrast, the HKW is placed together with the Liao Ning wolf, the geographically closest population to the Korean Peninsula, and closely related to other East Chinese wolves (Figure S4).

Altogether, these results suggest that the PZW is not originally from the Korean Peninsula, but instead it is closely related to highland wolves from the Tibetan Plateau. We hypothesise that this specimen was obtained by the Pyongyang Central Zoo via some other route (e.g., exchange with another zoo). Unfortunately, information about this sample is limited. Given the HKW likely represents the original Korean peninsula population and in light of the original questions of this study, for the remaining analyses, we focused mainly on this specimen.

### 3.2 Dog admixture patterns in the historical Korean wolf

**FIGURE 2:**
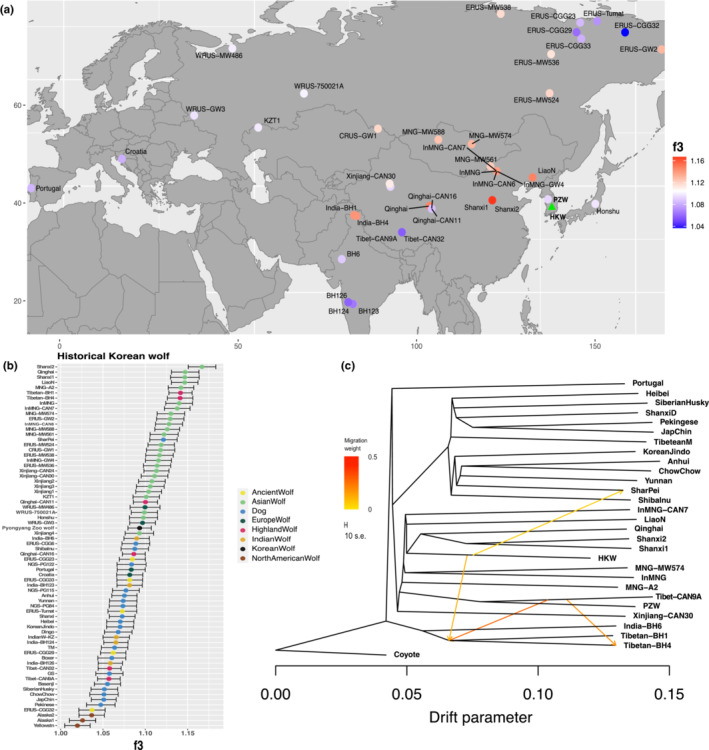
Genetic relationships and admixture patterns estimated by allele frequency data. (a) Outgroup *f*_3_‐statistics measuring shared genetic drift between the HKW and Eurasian wolves used in this study (*f*_3_ [HKW, Eurasian wolves; coyote]). Samples are shown in their approximate geographical locations. The samples with the highest *f*_3_‐statistics values are shown in red and the lowest in blue. HKW is represented in the map with a green triangle. Samples of zoo origin were excluded with the exception of the PZW. The coyotes were used as an outgroup. (b) Outgroup *f*_3_‐statistics showing levels of shared genetic drift between the HKW and wolves/dogs. Horizontal lines indicate 3 SEs estimated using a block‐jackknife procedure in 1 Mb blocks. (c) TreeMix admixture graph showing the result for four migration edges represented by arrows. The arrows indicate admixture events and their colour correlates with the intensity of the estimated gene flow between lineages. The analyses were performed on a SNP panel of 1,904,538 transversion sites. HKW, historical Korean wolf.

Next, we used outgroup *f*_3_ *‐*statistics to explore more deeply the identified relationships, by evaluating the amount of shared genetic drift between HKW, PZW and other wolf populations. The outgroup *f*_3_ *‐*statistics results for the PZW were in agreement with the clustering and phylogenetic analyses, showing high shared genetic drift with highland wolves from the Tibetan Plateau (Figure S5). The outgroup *f*_3_ *‐*statistics showed the HKW is most closely related to east Asian wolves, mainly from Central and East China, but also to the highland wolves BH1 and BH4, and the Shar Pei dog (Figure 2a,b). We then used TreeMix (Pickrell & Pritchard, 2012) to look into potential admixture in the HKW suggested by the outgroup *f*_3_ *‐*statistics results. This analysis was performed using a subset of samples, which includes the HKW and the closest Asian wolves as observed in the outgroup *f*_3_ *‐*statistics and NJ tree, as well as wolves representing relevant clades on the tree (Portuguese, Xinjiang‐CAN30, Indian‐BH6, Tibet‐CAN9A and the PZW), and Asian dog breeds. The overall recovered tree topology is in agreement with the NJ and ML trees, and the migration events estimated are in agreement with the admixture patterns observed before (Figure 2c; Figures S6 and S7). When allowing one to three migration events, we find the Tibetan wolves BH1 and BH4 are placed in the same clade as the Indian wolves, but appear to have had gene flow from the HKW lineage and highland wolves, confirming its admixed nature (Figures S6 and S7). The high level of admixture of these two Tibetan wolf samples from Ladakh was already mentioned in the original study where they were described (cf. Hennelly et al., 2021). Finally, the treemix admixture graph shows an admixture event between HKW and the Shar Pei dog, when allowing for an additional migration edge, supporting our outgroup *f*_3_ *‐*statistics results (Figure 2c; Figures S6 and S7).

**FIGURE 3:**
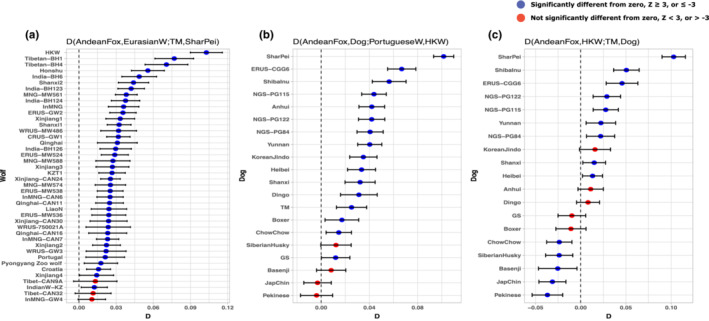
Gene flow between wolves and dog breeds. (a) *D*‐statistics testing for admixture between wolves and Shar Pei dogs. (b, c) *D*‐statistics tests showing gene flow between the historical Korean wolf (HKW) and different dog breeds. In all analyses, the Andean fox was used as an outgroup, and the Tibetan mastiff breed (TM) was used as a representative for dogs. In all cases, the Shar Pei appears with the highest *D* values, indicating admixture with wolves. The HKW shows significant admixture with dogs, in particular with the Shar Pei breed.

To formally test for admixture between the HKW and dogs, we used *D‐*statistics. First, to test the possibility of wolf admixture in the Shar Pei breed as an explanation for the gene flow observed in the TreeMix tree, we implemented a test in the form *D* (Andean fox, Eurasian wolves; Tibetan mastiff, Shar Pei). If the Shar Pei dog is admixed with wolves, we expect that all wolves share significantly more alleles with the Shar Pei in comparison to the Tibetan mastiff. In contrast, if the Shar Pei dog is not admixed with wolves, we expect that only wolves carrying dog ancestry will share significantly more alleles with the Shar Pei dog. The results showed that Shar Pei dog shares more alleles than the Tibetan mastiff with most of the Eurasian wolves in the test, indicating that Shar Pei dog carries wolf admixture. The especially high *D*‐statistics value obtained when HKW in used in the test further suggests that HKW is the most likely source of wolf ancestry in the Shar Pei dog (Figure 3a).

Next, we tested whether the HKW also had admixture from the Shar Pei dog by computing a test of the form *D* (Andean fox, Dog; Portuguese wolf, HKW) (Figure 3b). If HKW carries admixture from the Shar Pei dog, we expect all dogs to share significantly more alleles with HKW in comparison to the Portuguese wolf. Conversely, if only the Shar Pei dog shares significantly more alleles with HKW, that would imply gene flow from the HKW into the Shar Pei dog. Similarly, a second test in the form *D* (Andean fox, HKW, Tibetan mastiff, Dog) was performed to explore the dog admixture with the HKW (Figure 3c). In this case, if the HKW had admixed with Shar Pei, we expect most of the dogs to share significantly more alleles with the HKW, but if the Shar Pei dog is admixed with Korean wolves, just the Shar Pei dog should share significantly more alleles with the HKW in comparison to the Tibetan mastiff. The first test confirmed that the HKW is admixed with dogs, and the highest value in the analysis was obtained by the Shar Pei dog, suggesting that this breed is the main genetic source of the HKW admixture with dogs (Figure 3b). The result of the second test supported the admixture patterns observed before, where the Shar Pei dog is the breed with the highest obtained value (Figure 3c). Overall, *D‐*statistics and treemix graph results suggest there has been bi‐directional gene flow between the HKW and Shar Pei lineages.

### 3.3 Korean wolf's genetic diversity

**FIGURE 4:**
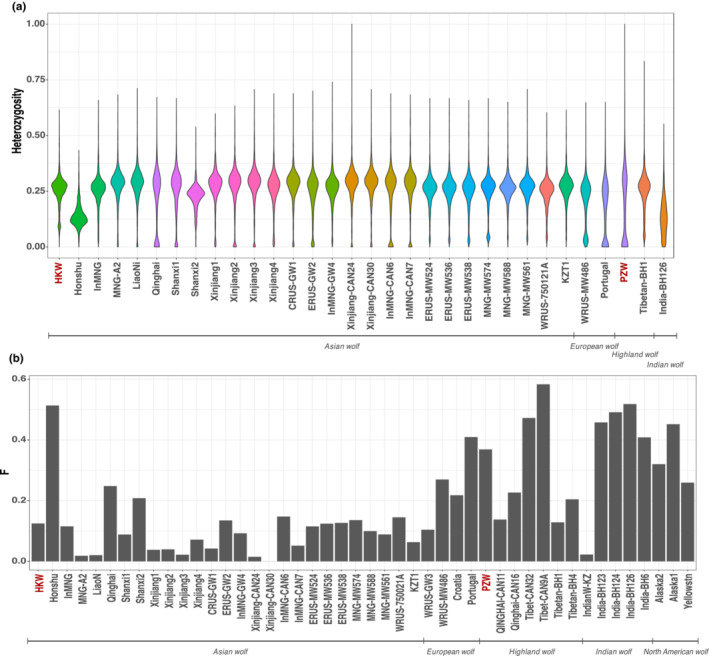
Assessing HKW genetic diversity. (a) Heterozygosity estimated in 1 Mb genomic windows for Asian wolves and representative European, Indian and Highland wolves. The HKW displays similar heterozygosity to most of the compared wolf individuals, but it shows a proportion of low heterozygosity regions. (b) Inbreeding coefficients (*F*) estimated for all modern and historical wolves in our dataset. HKW has a low inbreeding value (0.12) when compared to the rest of the wolf samples. Both analyses were performed on genotype imputed data. HKW, historical Korean wolf.

Given the decline of Korean wolf populations and the newly obtained evidence of admixture with the Shar Pei dog, we measured the genetic diversity of the HKW to gain insights into the demographic changes affecting these populations. We estimated heterozygosity in non‐overlapping windows of 1 Mb across the first 704 scaffolds longer than 1 Mb, using a subset of relevant wolf samples including the HKW and the PZW. When comparing heterozygosity, it is clear that, on average, HKW presented similar levels of heterozygosity to the rest of the wolves, even though there is a slight tendency to lower heterozygous regions (Figure 4a). Estimated heterozygosity based on genotype‐likelihoods showed similar values and patterns (Figure S8). Complementary to this analysis, we estimated the inbreeding coefficient (*F*) of all the modern wolves in our dataset. Our results show low inbreeding levels (0.12) for the HKW compared to other wolves (Figure 4b).

## 4 DISCUSSION

Our results demonstrate that Korean wolves are closely related to East Chinese wolf populations and do not seem to be highly genetically differentiated. This implies that wolf populations from the Korean Peninsula were not geographically isolated from the rest of the continental populations, as was suggested by Abe, who argued that the Yalou River could serve as a geographical barrier (Abe, 1936). Previously published data by Ishiguro et al. (2009, 2010) indicated similar results when analysing the mitochondrial control region. For this reason, and despite the limitation of having used just one wolf sample originally from Korea in this study, we suggest that the observed association patterns correctly reflect the genetic relationships of the likely vanished Korean wolf populations. Therefore, from a genomic perspective, there is no evidence to separate Korean wolves from other closely related populations from China, Mongolia, and Russia currently belonging to the *Canis lupus lupus* subspecies.

Surprisingly, in some analyses, we observed a higher affinity between the HKW and wolves from as far as Qinghai or Ladakh than to wolves from Mongolia or Inner Mongolia that are geographically closer. Our results show regional population structure among the East Asian wolves, where the populations from Inner Mongolia, Mongolia, and East Russia form one group, and the wolves from Korea, Shanxi, Liao Ning, and Qinghai form a second group. Furthermore, the gene flow observed between the HKW and highland wolf populations from Ladakh in the north of India emphasises such population structure. This suggests that wolf mobility and gene flow between central China or even western Asian regions and eastern China and the Korean Peninsula is more plausible than the mobility to northern regions. Considering the high dispersal capacity of wolves, in the case of the Korean populations, the reduced gene flow with wolves from Inner Mongolia and Mongolia, or East Russia, is probably not related to topographic barriers like the Mongolian plateau, the Great Khingan Mountains, the Lesser Khingan Mountains, or the Changbai Mountains, but to ecological affinities. It has been observed before the existence of significant isolation by distance among neighbouring wolf populations (Geffen et al., 2004), which has been associated with ecological variables such as vegetation type, temperature, or even snow cover, among others (Geffen et al., 2004; Pilot et al., 2006). These habitat preferences have been explained in relationship to prey specialisation, due to some of these environmental factors defining the ungulate communities that are the wolves' main prey (Carmichael et al., 2001; Mech & Boitani, 2003; Musiani et al., 2007; Pilot et al., 2006). Similarly, a north–south differentiation pattern among wolf populations has been described before, again, associated with changes in environmental variables (Randi et al., 2001; Stronen et al., 2013). Therefore, the grasslands of the Mongolian plateau, or the denser coniferous forests of Russia, as well as the colder weather and other environmental variables associated with those ecosystems could represent less familiar or suitable habitats for Korean wolf populations, it being more preferable for them to disperse through the North China Plain to the west. In order to corroborate this possible gene flow corridor, it will be important to increase the number of Chinese wolf genomes from regions that are not yet well represented.

Our analyses show that the PZW is most likely a highland wolf due to its strong genetic affinity with other highland wolves from the Tibetan plateau. The origin of this wolf is uncertain to us. It is possible that the PZW was considered equivalent to the Korean wolf from a taxonomic point of view since, historically, Korean wolves were classified as the subspecies *C. l. chanco*, the same subspecies that has been used to describe highland wolves from the Tibetan plateau. If that is the case, our results suggest that Korean wolf conservation programmes would not benefit from including the PZW.

Our results are in line with previous studies showing that isolated and diminished wolf populations have a higher chance of hybridising with closely related species, especially dogs (Ballard et al., 2003; Ciucani et al., 2022; Niemann et al., 2021; Phillips et al., 2003; Randi et al., 2001; vonHoldt et al., 2022). Our data revealed that the HKW is admixed with dogs, with the Shar Pei breed being the most likely source of ancestry contributing to the genetic composition of this specimen. The relatively high estimated heterozygosity levels across the genome of the HKW, as well as its low inbreeding coefficient estimation, could be explained by admixture with dogs. We could expect to find low heterozygosity levels and inbreeding in populations in decline, as can be observed in the case of the Honshu wolf from Japan, which suffered a similar history of decrease in population size and subsequent extinction (Ishiguro et al., 2009; Matsumura et al., 2014; Niemann et al., 2021). Strong admixture signatures are more common in small and isolated populations, which could be the situation for Korean wolves. However, there is a possibility that the admixture pattern found here represents a particular case of a wolf individual with higher or more recent dog admixture than the average in the Korean wolf populations. To truly elucidate the extent and frequency of dog admixture in the wolf populations from the Korean Peninsula, it is necessary to expand the analysis to a larger number of historical specimens from across this region.

In the same way, our results indicate that the Chinese Shar Pei breed is admixed with wolves, mainly from Asia. Taking into consideration that Shar Pei was close to extinction during the 20th century due to its prohibition after the Chinese Communist Revolution (The Editors of Encyclopedia Britannica, 2023), this breed likely went through a population bottleneck, reducing its genetic diversity and favouring admixture with wolves. According to historical records about the Shar Pei dog, the breed survived in Taiwan and Hong Kong, making it unlikely that the main wolf source of admixture was wolves from Korea. Therefore, we consider that the high affinity between the HKW and the Shar Pei dog observed in the *D‐*statistics is due to the Shar Pei ancestry in the Koren wolf rather than the other way around.

Our findings have the potential to contribute to wolf conservation strategies, both at a local level for the reintroduction of wolves in the Korean Peninsula and at a regional level. In the context of the Korean Peninsula, clarifying the genetic affinities of Korean wolves could allow the definition of an appropriate genetic pool for wolf populations used in conservation and reintroduction programmes. Our preliminary results suggest wolves from neighbouring regions to the Korean Peninsula, such as northern China, would be the best option for replacing the extirpated Korean populations. Perhaps less desirable could be the use of populations from the Mongolian Plateau or eastern Russia for this purpose; nevertheless, further historical samples from Korean wolves would be necessary to confirm it. At a regional level, the detection, protection or creation of biological corridors that allow the dispersion and gene flow of wolf populations in central and eastern China, connecting with the Korean Peninsula, would be ideal for the protection of the species. These corridors would have the potential to also benefit other threatened species including prey species.

## AUTHOR CONTRIBUTIONS

**Germán Hernández‐Alonso:** Conceptualization (lead); formal analysis (lead); investigation (lead); visualization (lead); writing – original draft (lead). **Jazmin Ramos‐Madrigal:** Formal analysis (supporting); methodology (supporting); supervision (supporting); writing – original draft (supporting). **Xin Sun:** Formal analysis (supporting); methodology (supporting). **Camilla Hjorth Scharff‐Olsen:** Data curation (lead). **Mikkel‐Holger S. Sinding:** Data curation (supporting); writing – review and editing (supporting). **Nuno F. Martins:** Data curation (supporting); writing – review and editing (supporting). **Marta Maria Ciucani:** Data curation (supporting); writing – review and editing (supporting). **Sarah S. T. Mak:** Data curation (supporting); writing – review and editing (supporting). **Liam Thomas Lanigan:** Data curation (supporting); writing – review and editing (supporting). **Cecilie G. Clausen:** Data curation (supporting); writing – review and editing (supporting). **Jong Bhak:** Data curation (supporting); resources (lead); writing – review and editing (supporting). **Sungwon Jeon:** Data curation (supporting); resources (supporting); writing – review and editing (supporting). **Changjae Kim:** Data curation (supporting); resources (supporting); writing – review and editing (supporting). **Kyung Yeon Eo:** Resources (supporting); writing – review and editing (supporting). **Seong‐Ho Cho:** Resources (supporting); writing – review and editing (supporting). **Bazartseren Boldgiv:** Resources (supporting); writing – review and editing (supporting). **Gankhuyag Gantulga:** Resources (supporting); writing – review and editing (supporting). **Zunduibaatar Unudbayasgalan:** Resources (supporting); writing – review and editing (supporting). **Pavel A. Kosintsev:** Resources (supporting); writing – review and editing (supporting). **Hans K. Stenøien:** Funding acquisition (supporting); writing – review and editing (supporting). **M. Thomas P. Gilbert:** Funding acquisition (lead); resources (supporting); supervision (supporting); writing – review and editing (lead). **Shyam Gopalakrishnan:** Supervision (supporting); writing – review and editing (supporting).

## BENEFIT‐SHARING STATEMENT

The research work presented here was the result of an international collaboration that involved the participation and co‐authorship of scientists from the different countries providing the wolf samples. The results produced by this collaboration can directly impact on the local and regional conservation strategies of wolf populations.

## Supporting information


Appendix S1

## Data Availability

Generated raw sequence reads are deposited to the European Nucleotide Archive (Project ID: PRJEB57591).
