# Correction to “Conservation Implications of Elucidating the Korean Wolf Taxonomic Ambiguity Through Whole‐Genome Sequencing”

**DOI:** 10.1002/ece3.71393

**Published:** 2025-06-18

Hernández‐Alonso, G., Ramos‐Madrigal, J., Sun, X., Scharff‐Olsen, C. H., Sinding, M.‐H., Martins, N. F., Ciucani, M. M., Mak, S. S. T., Lanigan, L. T., Clausen, C. G., Bhak, J., Jeon, S., Kim, C., Eo, K. Y., Cho, S.‐H., Boldgiv, B., Gantulga, G., Unudbayasgalan, Z., Kosintsev, P. A. … Gopalakrishnan, S. (2023). Conservation implications of elucidating the Korean wolf taxonomic ambiguity through whole‐genome sequencing. *Ecology and Evolution*, 13, e10404. https://doi.org/10.1002/ece3.10404

The following reference is in preprint version:

Ciucani, M. M., Ramos‐Madrigal, J., Hernández‐Alonso, G., Carmagnini, A., Aninta, S. G., Scharff‐Olsen, C. H., Lanigan, L. T., Fracasso, I., Clausen, C. G., Aspi, J., Kojola, I., Baltrūnaitė, L., Balčiauskas, L., Moore, J., Åkesson, M., Saarma, U., Hindrikson, M., Hulva, P., Bolfíková, B. Č., … Gopalakrishnan, S. (2022). Genomes of the extinct Sicilian wolf reveal a complex history of isolation and admixture with ancient dogs. *bioRxiv*. https://doi.org/10.1101/2022.01.21.477289

It should be updated to the peer‐reviewed version of the article:

Marta Maria Ciucani, Jazmín Ramos‐Madrigal, Germán Hernández‐Alonso, Alberto Carmagnini, Sabhrina Gita Aninta, Xin Sun, Camilla Hjorth Scharff‐Olsen, Liam Thomas Lanigan, Ilaria Fracasso, Cecilie G. Clausen, Jouni Aspi, Ilpo Kojola, Laima Baltrūnaitė, Linas Balčiauskas, Jane Moore, Mikael Åkesson, Urmas Saarma, Maris Hindrikson, Pavel Hulva, Barbora Černá Bolfíková, Carsten Nowak, Raquel Godinho, Steve Smith, Ladislav Paule, Sabina Nowak, Robert W. Mysłajek, Sabrina Lo Brutto, Paolo Ciucci, Luigi Boitani, Cristiano Vernesi, Hans K. Stenøien, Oliver Smith, Laurent Frantz, Lorenzo Rossi, Francesco Maria Angelici, Elisabetta Cilli, Mikkel‐Holger S. Sinding, M. Thomas P. Gilbert, Shyam Gopalakrishnan. The extinct Sicilian wolf shows a complex history of isolation and admixture with ancient dogs. *iScience*, Volume 26, Issue 8, 2023, 107307, ISSN 2589‐0042, https://doi.org/10.1016/j.isci.2023.107307

We apologize for this error.

