# Supplementary material for: Conservation implications of elucidating the Korean wolf taxonomic ambiguity through whole‐genome sequencing
Source: Ecol Evol. 2023 Aug 4;13(8):e10404. doi: 10.1002/ece3.10404 (PMC10401669; doi:10.1002/ece3.10404)
Supplement: Supplementary file 1 — Appendix S1 [file ECE3-13-e10404-s001.pdf]

Supplemental Information for:

Conservation implications of elucidating the Korean wolf taxonomic ambiguity through whole-genome sequencing

Germán Hernández-Alonso, Jazmín Ramos-Madrigal, Xin Sun, Camilla Hjorth Scharff-Olsen, Mikkel-Holger S. Sinding, Nuno F. Martins, Marta Maria Ciucani, Sarah S. T. Mak, Liam Thomas Lanigan, Cecilie G. Clausen, Jong Bhak, Sungwon Jeon, Changjae Kim, Kyung Yeon Eo, Seong-Ho Cho, Bazartseren Boldgiv, Gankhuyag Gantulga, Zunduibaatar Unudbayasgalan, Pavel A. Kosintsev, Hans K. Stenøien, M. Thomas P. Gilbert, Shyam Gopalakrishnan

Table S1. Resequenced wolf samples.

**Project ID**	**Museum ID**	**Date**	**Museum of origin**	**Species**	**Country**	**Sex**	**Tissue**	**Average whole genome coverage**
Pyongyang Zoo wolf	IN149	2005	Seoul Grand Park, South Korea	*Canis lupus*	South Korea	Female	Blood	25.2989
Historical Korean wolf	/	Before 1945	Kyungpook University Museum, Seoul, South Korea	*Canis lupus*	South Korea	Female	Skin	7.2511
WRUS-MW486	V25-RU	2000	Mikael Åkesson & Jouni Aspi (SLU)	*Canis lupus*	Russia	Male	Skin	5.811174736
ERUS-MW524	3	2015	Morten Skage (Oslo, Norway)	*Canis lupus*	Russia	Female	Extracts	5.784092198
ERUS-MW536	15	2015	Morten Skage (Oslo, Norway)	*Canis lupus*	Russia	Female	Extracts	5.732749185
ERUS-MW538	17	2015	Morten Skage (Oslo, Norway)	*Canis lupus*	Russia	Male	Extracts	5.850073506
MNG-MW574	24	2019	Boldgiv Bazartseren	*Canis lupus*	Mongolia	Female	Cheek tissue	5.770702451
MNG-MW588	43	2019	Boldgiv Bazartseren	*Canis lupus*	Mongolia	Female	Fresh skin	5.495638571
MNG-MW561	4	2018	Boldgiv Bazartseren	*Canis lupus*	Mongolia	Female	Muscle tissue	5.481997786
WRUS-750021A	750021	modern	Yekaterinburg Museum	*Canis lupus*	Russia	Female	Muscle tissue	5.460293376
KZT1	750115	modern	Yekaterinburg Museum	*Canis lupus*	Kazakhstan	Male	Muscle tissue	6.431657396
MNG-A2	A70094	Birth 25/3/2007	Zoo Zürich	*Canis lupus*	Germany	Male	Muscle tissue	7.64

Table S2. Whole samples dataset.

Project ID	Species	Location	Coverage	Sample	SAMN/SRA or Accession/Project numbers	Publication/Source
Pyongyang Zoo wolf	Wolf	Korea	25.2989	KoreanWolf	BioSample: SAMEA113937679; Accession: ERS15931840	this study
Historical Korean wolf	Wolf	Korea	1.09618	HistoricKoreanWolf	BioSample: SAMEA113937680; Accession: ERS15931841	this study
Yellowstn	Wolf	USA	24.292	Yellowstone2Wolf	BioSample: SAMN10174954; Sample name: Yellowstone2_wolf; SRA: SRS3887573	vonHoldt et al. 2016
Alaska1	Wolf	Alaska	11.8458	StLawrenceIsland	BioSample: SAMN10246094; Sample name: GreyWolf_StLawrenceIsland; SRA: SRS3941477	Sinding et al. 2018
Alaska2	Wolf	Alaska	13.5781	PacificCoast	BioSample: SAMN10246091; Sample name: GreyWolf_PacificCoast; SRA: SRS3941476	Sinding et al. 2018
Croatia	Wolf	Croatia	6.25096	Novembre_Croatian_Wolf	BioSample: SAMN03366712; Sample name: RKW3919; SRA: SRS1025420	Freedman et al. 2014
Portugal	Wolf	Portugal	22.8624	PortugueseWolf	BioSample: SAMN02921316; Sample name: ptw; SRA: SRS661492	Fan et al. 2016
Honshu	Wolf	Japan	3.94849	1886_1_Honshu	BioSample: SAMEA7615946; SRA: ERS5374233	Niemann et al. 2021
InMNG	Wolf	China	4.75869	InnerMongoliaWolf	BioSample: SAMN03168393; Sample name: LUPZCHN00002; SRA: SRS1135621	Wang et al. 2016
MNG-A2	Wolf	Mongolia	7.64687	Mongolian_wolf_A2	BioSample: SAMEA113937690; Accession: ERS15931851	this study
CRUS-GW1	Wolf	Altai, Russia	9.57791	GW1	BioSample: SAMN01974486; Sample name: GW1; SRA: SRS402038	Wang et al. 2013
ERUS-GW2	Wolf	Russia	7.73573	GW2	BioSample: SAMN01974487; Sample name: GW2; SRA: SRS402061	Wang et al. 2013
WRUS-GW3	Wolf	Russia	9.83913	GW3	BioSample: SAMN01974488; Sample name: GW3; SRA: SRS402066	Wang et al. 2013
InMNG-GW4	Wolf	Inner Mongolia, China	7.87839	GW4	BioSample: SAMN01974489; Sample name: GW4; SRA: SRS401789	Wang et al. 2013
LiaoN	Wolf	China	12.8896	LiaoNingWolf	BioSample: SAMN03168394; Sample name: LUPWCHN00003; SRA: SRS1135629	Wang et al. 2016
Qinghai	Wolf	China	24.893	QinghaiWolf	BioSample: SAMN10174946; Sample name: Qinghai_wolf; SRA: SRS3887562	vonHoldt et al. 2016
Shanxi1	Wolf	China	10.5221	Shanxi1Wolf	BioSample: SAMN03168396; Sample name: LUPZCHN00006; SRA: SRS1135626	Wang et al. 2016
Shanxi2	Wolf	China	2.71456	Shanxi2Wolf	BioSample: SAMN03168395; Sample name: LUPZCHN00005; SRA: SRS1135620	Wang et al. 2016
Xinjiang1	Wolf	China	8.71962	Xinjiang1Wolf	BioSample: SAMN03168397; Sample name: LUPWCHN00008; SRA: SRR2827601	Wang et al. 2016
Xinjiang2	Wolf	China	14.2893	Xinjiang2Wolf	BioSample: SAMN03168399; Sample name: LUPWCHN00010; SRA: SRR2827602	Wang et al. 2016
Xinjiang3	Wolf	China	25.2979	Xinjiang3Wolf	BioSample: SAMN03168398; Sample name: LUPWCHN00009; SRA: SRR2827608	Wang et al. 2016
Xinjiang4	Wolf	China	11.953	Xinjiang4Wolf	BioSample: SAMN03168400; Sample name: LUPWCHN00013; SRA: SRR2827611	Wang et al. 2016
Qinghai-CAN11	Wolf	China	22.904	Chinese_CAN11	ProjectNumber: PRJNA448733	Zhang et al. 2014
Qinghai-CAN16	Wolf	China	23.2029	Chinese_CAN16	ProjectNumber: PRJNA448734	Zhang et al. 2014
Xinjiang-CAN24	Wolf	China	21.0567	Chinese_CAN24	ProjectNumber: PRJNA448735	Zhang et al. 2014
Xinjiang-CAN30	Wolf	China	23.5947	Chinese_CAN30	ProjectNumber: PRJNA448736	Zhang et al. 2014
Tibet-CAN32	Wolf	China	22.2005	Chinese_CAN32	ProjectNumber: PRJNA448739	Zhang et al. 2014
InMNG-CAN6	Wolf	China	22.3432	Chinese_CAN6	ProjectNumber: PRJNA448737	Zhang et al. 2014
InMNG-CAN7	Wolf	China	19.4338	Chinese_CAN7	ProjectNumber: PRJNA448738	Zhang et al. 2014
Tibet-CAN9A	Wolf	China	23.0481	Chinese_CAN9A	ProjectNumber: PRJNA448740	Zhang et al. 2014

IndianW-KZ	Wolf	India	37.82	IndiaWolf	BioSample: SAMN02921311; Sample name: inw; SRA: SRS661487	Fan et al. 2016
WRUS-MW486	Wolf	Russia	5.69880796	MW486	BioSample: SAMEA113937681; Accession: ERS15931842	this study
ERUS-MW524	Wolf	Russia	5.61864211	MW524	BioSample: SAMEA113937682; Accession: ERS15931843	this study
ERUS-MW536	Wolf	Russia	5.57929343	MW536	BioSample: SAMEA113937683; Accession: ERS15931844	this study
ERUS-MW538	Wolf	Russia	5.70093059	MW538	BioSample: SAMEA113937684; Accession: ERS15931845	this study
MNG-MW574	Wolf	Mongolia	5.60742916	MW574	BioSample: SAMEA113937685; Accession: ERS15931846	this study
MNG-MW588	Wolf	Mongolia	5.25760267	MW588	BioSample: SAMEA113937686; Accession: ERS15931847	this study
MNG-MW561	Wolf	Mongolia	5.25162299	MW561	BioSample: SAMEA113937687; Accession: ERS15931848	this study
WRUS-750021A	Wolf	Russia	5.67809279	750021A	BioSample: SAMEA113937688; Accession: ERS15931849	this study
KZT1	Wolf	Kazakhstan	6.18716953	750115A	BioSample: SAMEA113937689; Accession: ERS15931850	this study
BH123	Wolf	India	4.87204849	BH123_Indian_Wolf	BioSample: SAMN19655087; Sample name: BH123; SRA: SRS9175420	Hennelly et al. 2021
BH124	Wolf	India	6.592202442	BH124_Indian_Wolf	BioSample: SAMN19655088; Sample name: BH124; SRA: SRS9175419	Hennelly et al. 2021
BH126	Wolf	India	7.84775658	BH126_Indian_Wolf	BioSample: SAMN19655087; Sample name: BH126; SRA: SRS9175421	Hennelly et al. 2021
BH6	Wolf	India	5.363461062	BH6_Indian_Wolf	BioSample: SAMN19655086; Sample name: BH6; SRA: SRS9175418	Hennelly et al. 2021
Tibetan-BH1	Wolf	India	4.549868696	BH1_Tibetan_Wolf	BioSample: SAMN19655084; Sample name: BH1; SRA: SRS9175416	Hennelly et al. 2021
Tibetan-BH4	Wolf	India	4.708613679	BH4_Tibetan_Wolf	BioSample: SAMN19655085; Sample name: BH4; SRA: SRS9175417	Hennelly et al. 2021
ERUS-Tumat	Ancient Wolf	Siberia, Russia	5.884	Tumat	BioSample: SAMEA104106512; SRA: ERS1770405	Ramos-Madrigal et al. 2021
ERUS-CGG23	Ancient Wolf	Siberia, Russia	4.66088	CGG23	BioSample: SAMN14210385; Sample name: CGG23; SRA: SRS6226019	Sinding et al. 2020
ERUS-CGG29	Ancient Wolf	Siberia, Russia	6.9043	CGG29	BioSample: SAMEA7538371; SRA: ERS5294828	Ramos-Madrigal et al. 2021
ERUS-CGG32	Ancient Wolf	Siberia, Russia	15.038	CGG32	BioSample: SAMEA7538369; SRA: ERS5294826	Ramos-Madrigal et al. 2021
ERUS-CGG33	Ancient Wolf	Siberia, Russia	16.2361	CGG33	BioSample: SAMEA7538370; SRA: ERS5294827	Ramos-Madrigal et al. 2021
ERUS-CGG6	Ancient Dog	Siberia, Russia	9.67821	CGG6	BioSample: SAMN14210384; Sample name: CGG6; SRA: SRS6226017	Sinding et al. 2020
NGS-PG115	Dog	New Guinea Singing Dog	6.07381	PG115	BioSample: SAMN02485601; Sample name: PG115; SRA: SRS520076	Auton et al. 2013
NGS-PG122	Dog	New Guinea Singing Dog	5.73586	PG122	BioSample: SAMN02485602; Sample name: PG122; SRA: SRS520077	Auton et al. 2013
NGS-PG84	Dog	New Guinea Singing Dog	8.53732	PG84	BioSample: SAMN02485603; Sample name: PG84; SRA: SRS520078	Auton et al. 2013
ShibaInu	Dog	Japan	31.7249	ShibaInuFemale	BioSample: SAMN21035413; Sample name: SHIB000003; SRA: SRS10307623	Kolicheski et al. 2017
Dingo	Dog	Australia	5.11229	RKW13760	BioSample: SAMN03366709; Sample name: RKW13760; SRA: SRS1025425	Freedman et al. 2014
TM	Dog	China (Tibetan Mastiff)	9.11817	TM	BioSample: SAMN01974493; Sample name: dogTM; SRA: SRS402077	Wang et al. 2013
Boxer	Dog	Germany	26.6486	BoxerDog	camFam3.1	Lindblad et al. 2005
GS	Dog	Germany	23.7987	GShepDog	BioSample: SAMN01974494; Sample name: dogGS; SRA: SRS402078	Marsden et al. 2016

KoreanJindo	Dog	Korea	38.8859	KoreanJindo	BioSample: SAMD00009664; SRA: DRS001112	kim et al. 2012
Basenji	Dog	Africa	5.327	BasenjiDog	BioSample: SAMN03366708; Sample name: RKW13764; SRA: SRS1025426	Freedman et al. 2014
Anhui	Dog	China	5.43465	AnhuiDog	BioSample: SAMN03168367; Sample name: FAMICHN00025; SRA: SRS1135599	Wang et al. 2016
Yunnan	Dog	China	13.4547	Yunnan1Dog	BioSample: SAMN03168366; Sample name: FAMICHN00023; SRA: SRS1135600	Wang et al. 2016
SharPei	Dog	China	5.47585	SharPei01	BioSample: SAMN05356426; Sample name: NGSDOG024; SRA: SRS1539503	Metzger et al. 2017
Shanxi	Dog	China	13.7662	Shanxi1Dog	BioSample: SAMN03168358; Sample name: FAMICHN00015; SRA: SRS1135606	Wang et al. 2016
Pekinese	Dog	China	56.9781	Pekingese	BioSample: SAMN03801676; Sample name: Pekingese01; SRA: SRS984793	Decker et al. 2015
JapChin	Dog	Japan	49.0978	JapaneseChin	BioSample: SAMN15625782; Sample name: CHIN000001; SRA: SRS7094452	Marchant et al. 2017
ChowChow	Dog	China	5.16064	ChowChow01	BioSample: SAMN03801657; Sample name: ChowChow01; SRA: SRS984784	Decker et al. 2015
Heibei	Dog	China	15.2985	HebeiDog	BioSample: SAMN03168356; Sample name: FAMICHN00012; SRA: SRS1135605	Wang et al. 2016
SiberianHusky	Dog	Siberia, Russia	58.4725	SiberianHusky01	BioSample: SAMN03801690; Sample name: SiberianHusky01; SRA: SRS984799	Decker et al. 2015
CaliforniaCoyote	Coyote	USA	26.6795	C._latrans_California_C106	BioSample: SAMN10174934; Sample name: California_coyote; SRA: SRS3887584	vonHoldt et al. 2016
Mexico	Coyote	Mexico	11.3432	C._latrans_Mexico_MSB.83943	BioSample: SAMN10180422; Sample name: Coyote_Mexico; SRA: SRS3929741	Gopalakrishnan et al. 2018
AndeanFox	Andean Fox	South America	10.5065	AndeanFox	BioSample: SAMN02487034; Sample name: Lcu2_Pastora; SRA: SRS523207	Auton et al. 2013

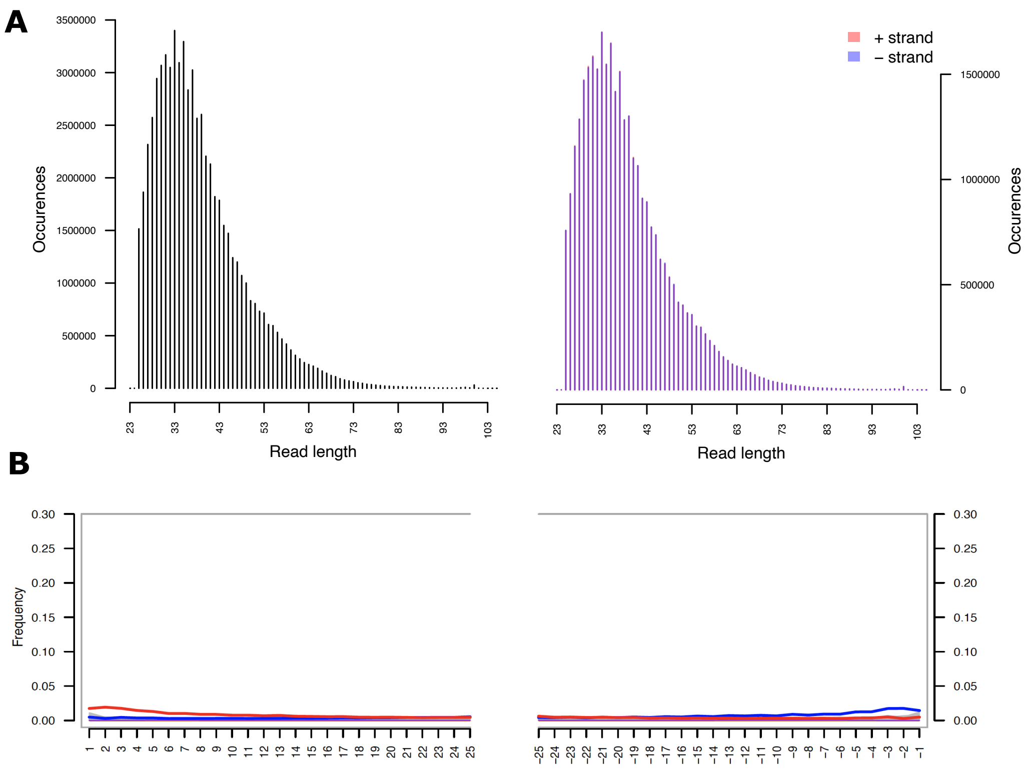

Figure S1. DNA damage assessment of historical Korean wolf data.
A) Sequence reads length distribution. Predominantly, short reads were obtained for the sequencing of the stuffed historical specimen. B) DNA damage pattern shown by the increase of C to T and G to A substitutions at the read ends. A low percentage of DNA damage can be observed in the historical Korean wolf sequenced reads.

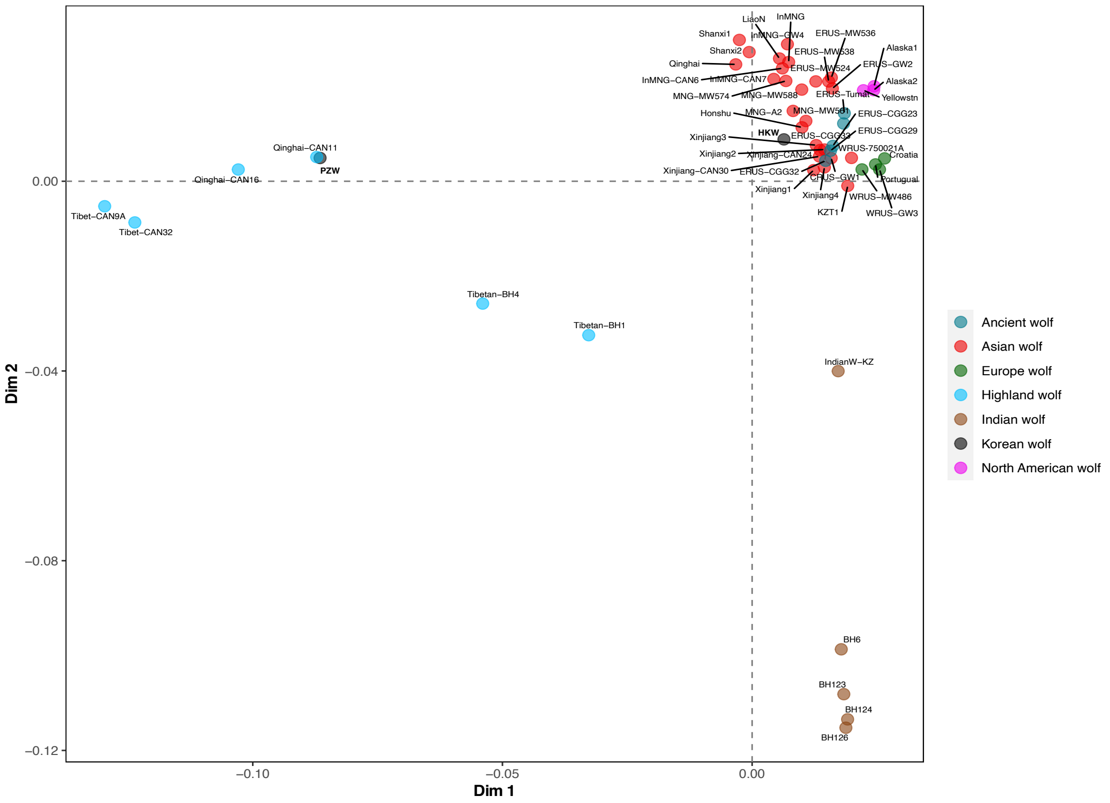

Figure S2. Extended MDS plot result. Related to Figure 1A.
MDS plot including just wolf genomes to explore in more detail the population structure among wolves. The analysis was performed using a pseudo-haploid dataset of 3,284,758 transversion sites.

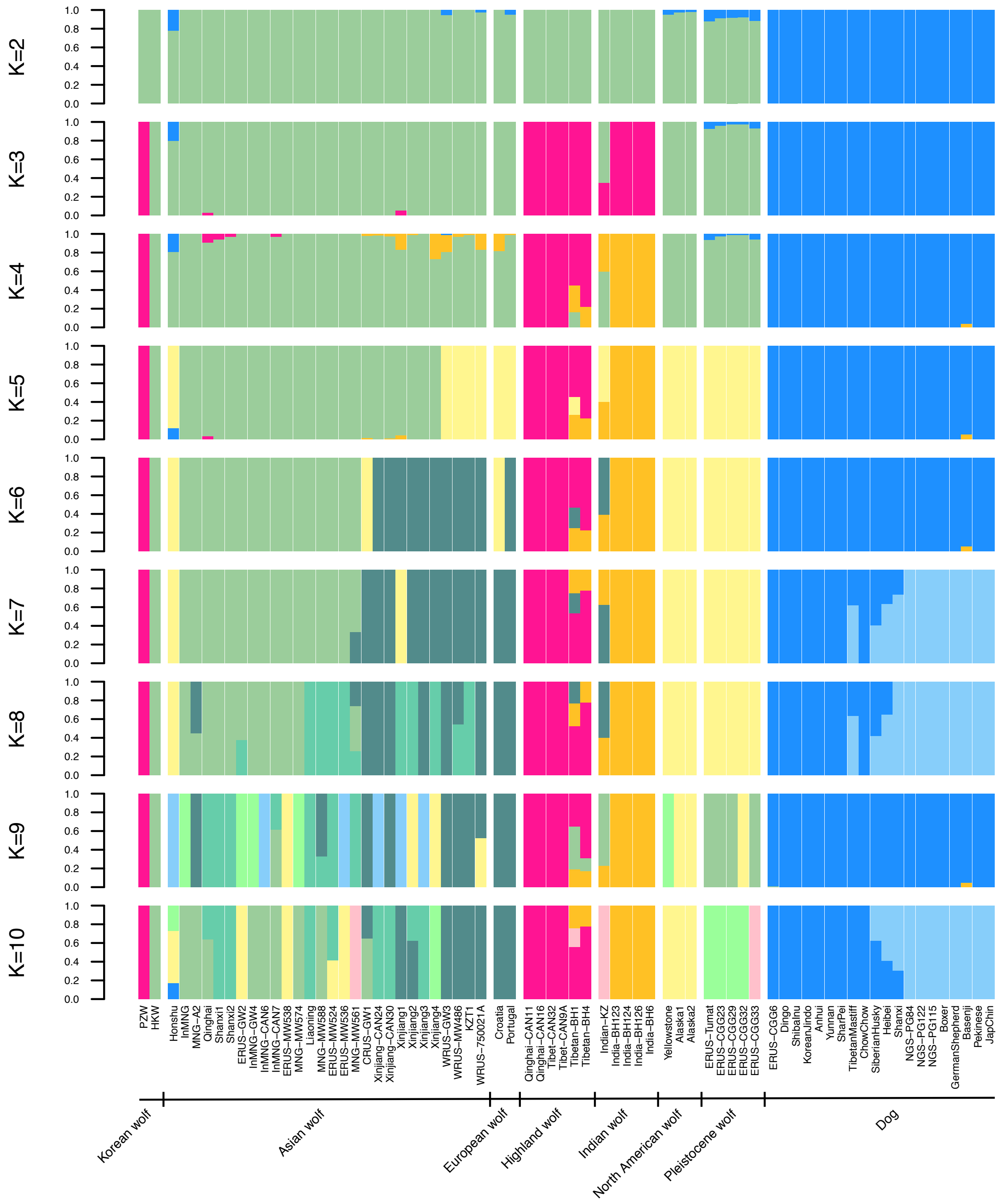

Figure S3. Extended admixture graphs estimated for 2 to 10 ancestry components (K). Related to Figure 1C.

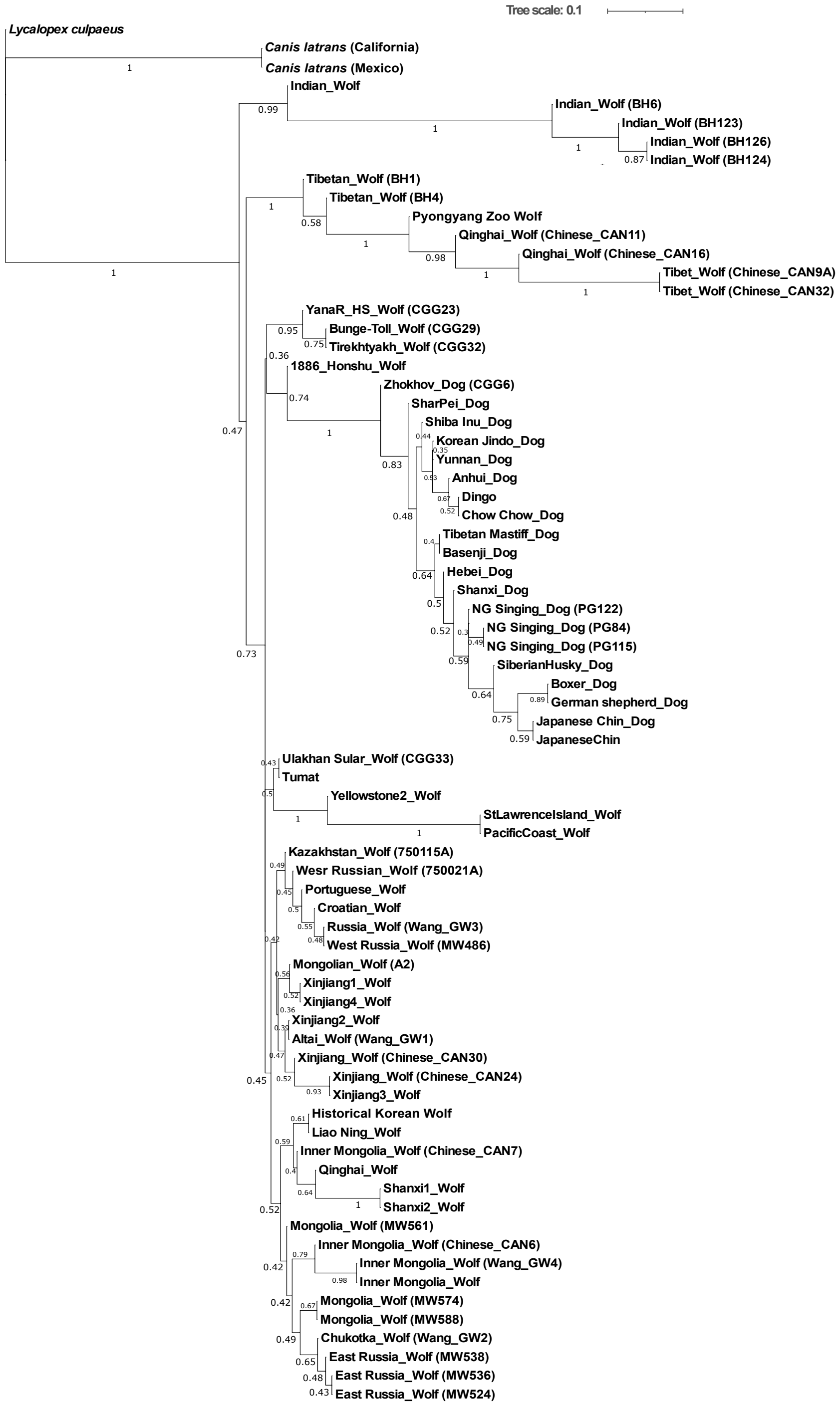

Figure S4. Phylogenetic relationships estimated by genomic data.
Maximum likelihood phylogeny built on 1000 concatenated trees. Bootstrap values are shown for each internal branch.

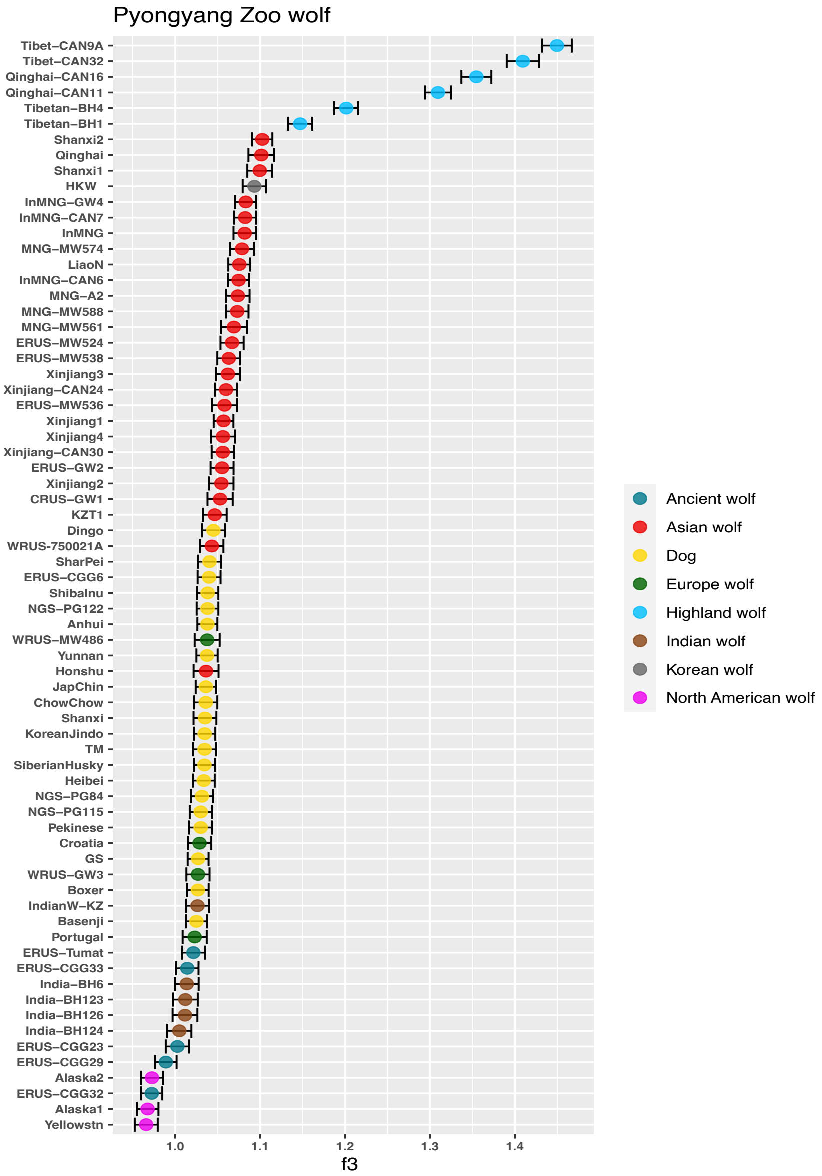

Figure S5. Assessment of shared genetic drift between the Pyongyang Zoo wolf and other wolves and dogs in the dataset.
To test the genetic affinities of the Pyongyang Zoo wolf we performed an outgroup *f3-statistics* analysis using the coyotes as outgroup.

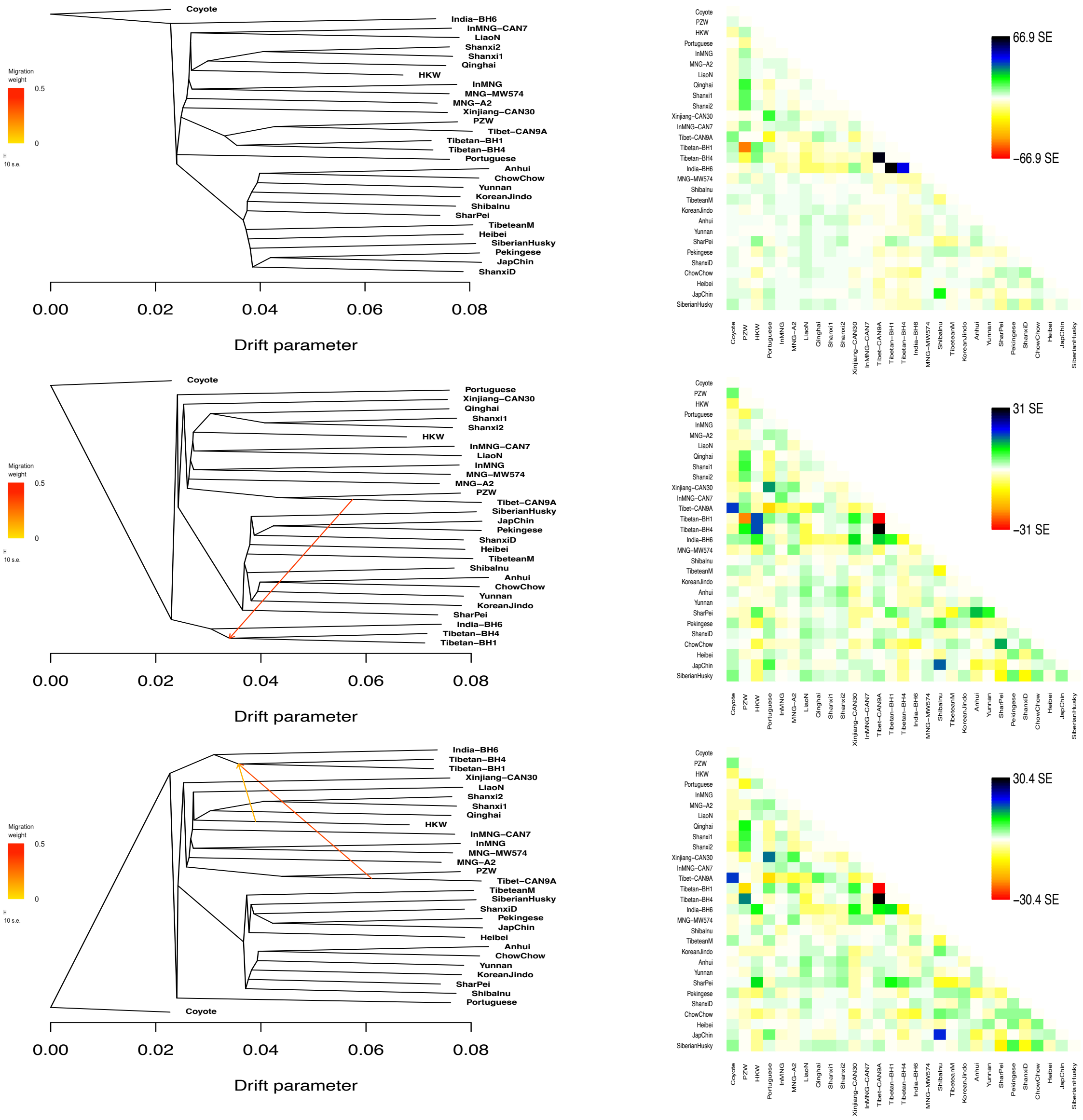

Figure S6. Extended Treemix admixture graphs and residual plots. Related to Figure 2C. Treemix graphs for 0 to 2 migration edges and residual plots from the fit of the model to the data. The analyses were run using an SNP panel of 1,904,538 transversion sites, excluding sites with missing data.

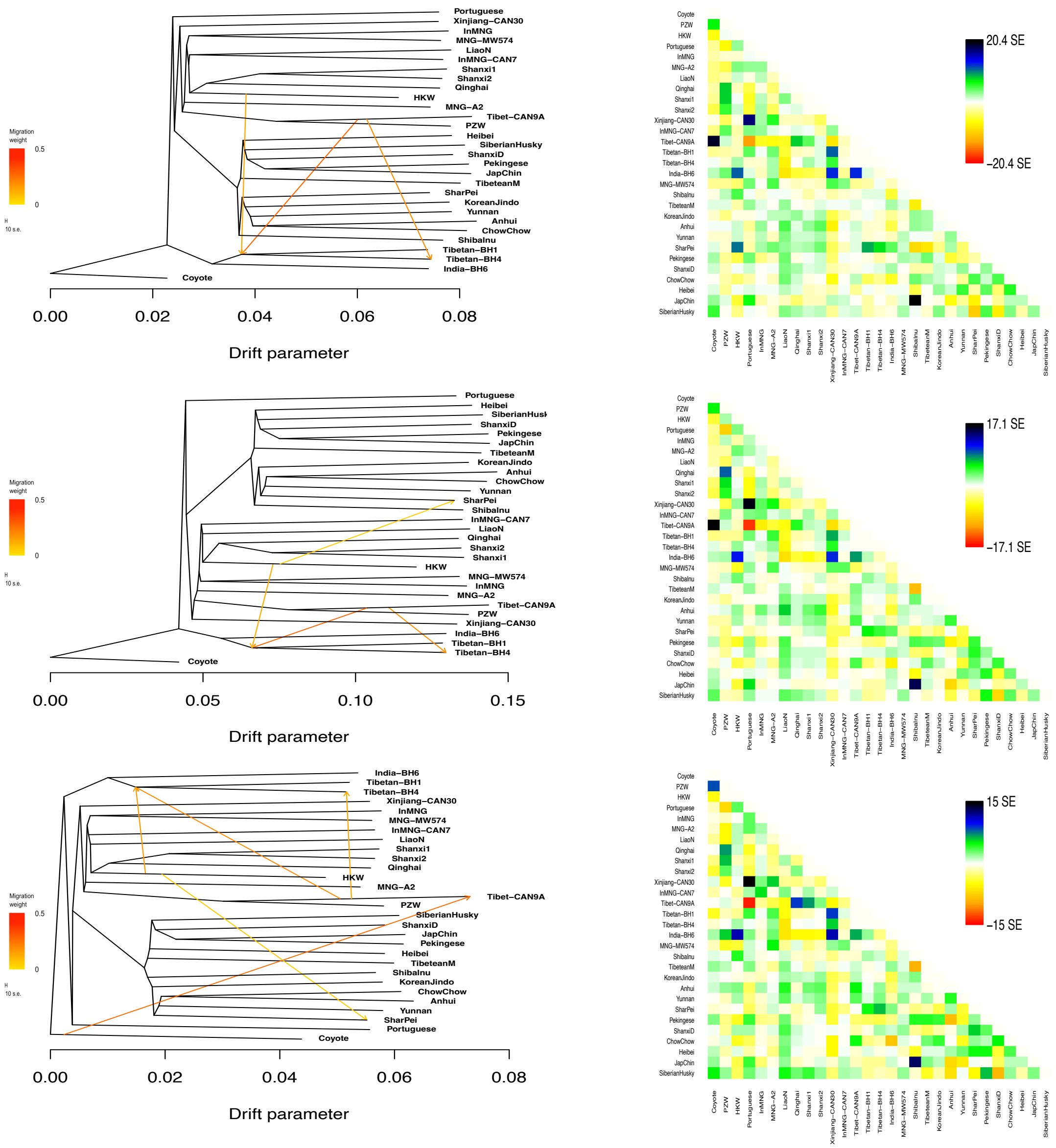

Figure S7. Extended Treemix admixture graphs and residual plots. Related to Figure 2C. Treemix graphs for 3 to 5 migration edges and residual plot from the fit of the model to the data.

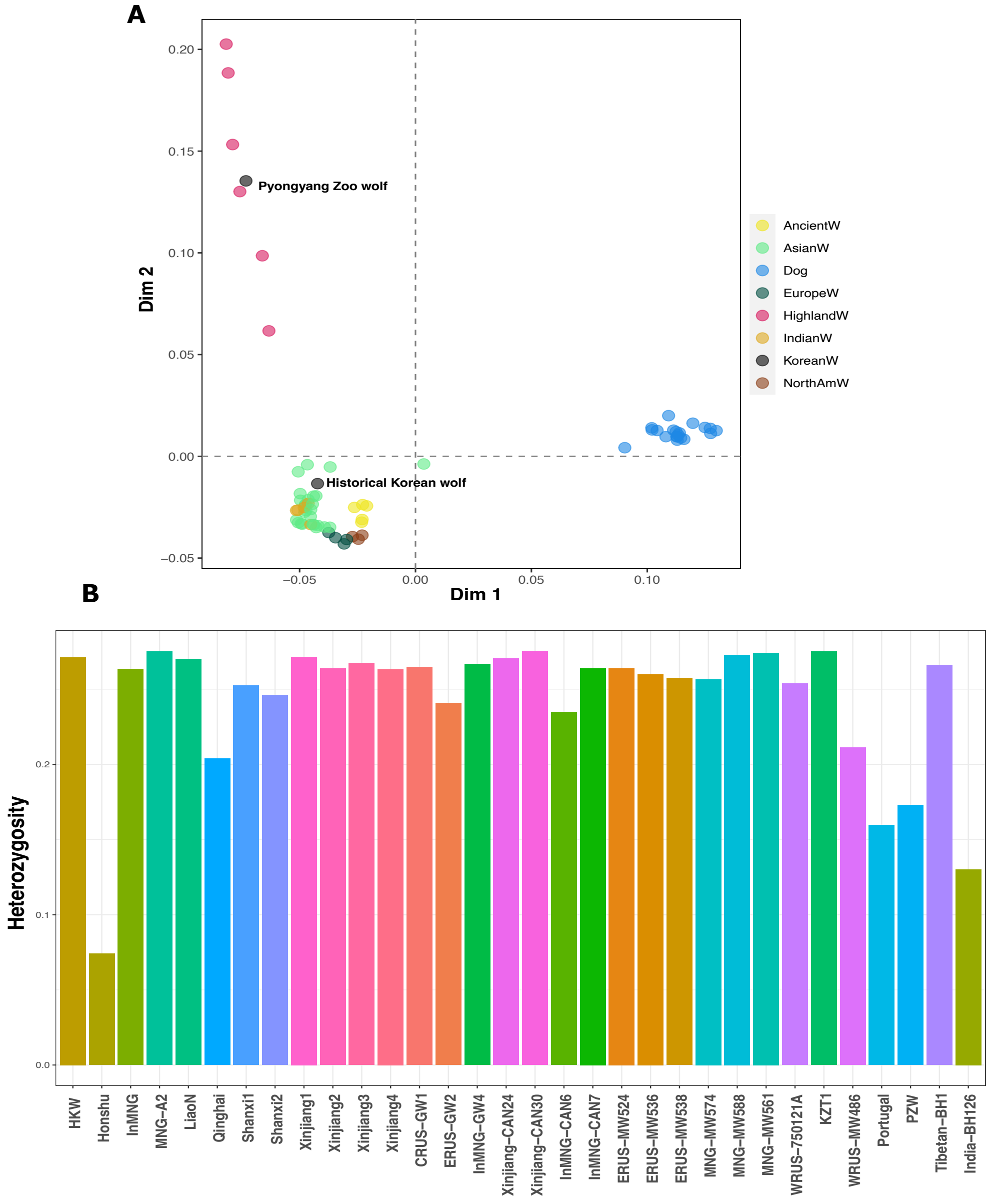

Figure S8. Analyses assessing the accuracy of imputed data.
A) MDS plot generated with the imputed data. The resulting structure closely resembles the patterns observed in the Figure 1A. B) Bar plot presenting estimated heterozygosity derived from genotype-likelihoods. The estimated values are consistent with the heterozygosity values obtained from the imputed data.

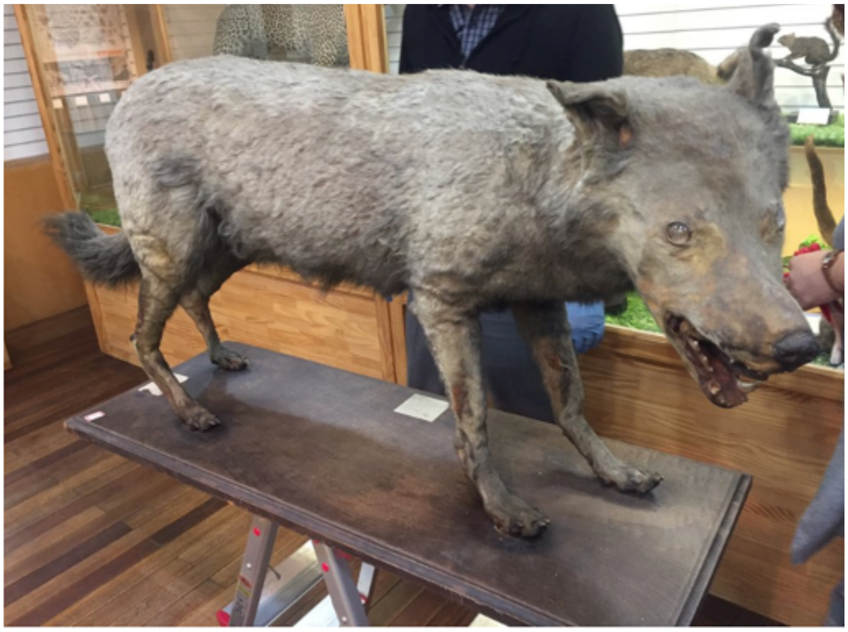

Figure S9. Stuffed historical Korean wolf specimen sequenced in this study.
